# A 14-Year-Old Patient with Chest Pain

**Published:** 2016

**Authors:** Maryam Hassanzad, Seyed Amir Mohajerani, Nima Hassanzad, Ali Akbar Velayati

**Affiliations:** 1 Pediatric Respiratory Diseases Research Center, National Research Institute of Tuberculosis and Lung Diseases (NRITLD), Shahid Beheshti University of Medical Sciences, Tehran, Iran

## WHAT IS YOUR DIAGNOSIS?

A 14-year-old boy was referred to our hospital with pain in his left posterior chest wall, cough, exertional dyspnea, weight loss, shoulder pain and inability to move his left hand. He had a history of chest trauma two weeks ago. During the past two weeks, he had two episodes of nausea and vomiting. During his admission to emergency department, his vital signs including blood pressure, heart rate, respiratory rate and oxygen saturation rate were normal. On physical examination, coarse crackles were auscultated in basal left lung and breath sounds were normal in other areas. Laboratory tests showed leukocytosis (WBC: 12.2×10^3^) and PLT 460×10^3^. CRP was positive (3+) and ESR was 96 mm/h. Sputum culture was also positive for Candida.

**Figure 1.**
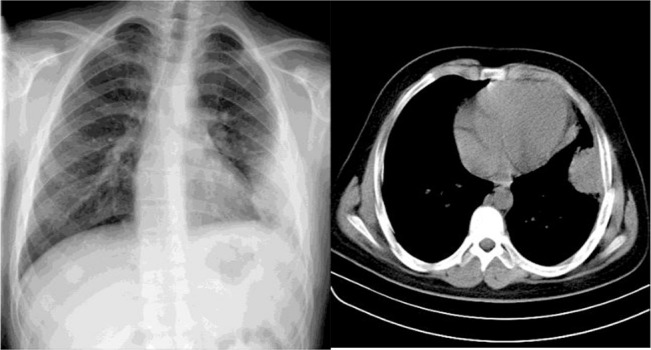
CXR shows a sub-pleural mass with silhouette sign. CT scan showed that there was a sub-pleural mass like consolidation in the LLL measuring up to 45×44mm associated with subtle ipsilateral pleural reaction. Right lung was intact.

**Figure 2.**
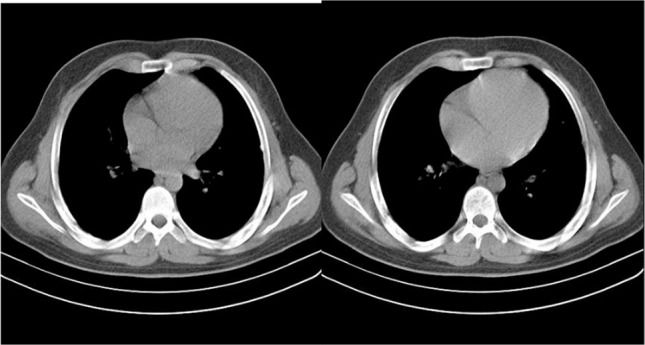
Chest CT one month (left) and three months (right) later

Chest X-radiography (CXR) showed that the patient had a left lower lobe (LLL) sub-pleural loculation. On chest computed tomography (CT), there was a subpleural mass like consolidation in LLL measured up to 45×44 mm associated with subtle ipsilateral pleural reaction. Right lung was intact (Figure 1 and 2). Ultrasound revealed a hypoecho mass of the size of 52×44 mm at inner part of the left lateral chest suggesting hematoma. One week later, the size of the mass decreased significantly to 43×36 mm on ultrasound.
