# Supplementary material for: A 14-Year-Old Patient with Chest Pain
Source: Tanaffos. 2016;15(4):249. (PMC5410123)
Supplement: Related texts

# Other: Diagnosis: The patient was diagnosed with subpleural hematoma as a complication of blunt trauma

**Published:** 2016

## TREATMENT

### Conservative Management or Thoracentesis

The patient was followed for one month without any interventions or thoracentesis. The patient’s sub-pleural hematoma was absorbed after one month spontaneously. One month later, ultrasound showed that sub-pleural hematoma mass was completely absorbed. Follow-up chest CT confirmed that sub-pleural hematoma had been absorbed with no signs of any remnant hematoma.

Thoracic trauma has an overall fatality rate of 10.1%, which is the highest in patients with cardiac or tracheobronchial-esophageal injuries. Sub-pleural hematoma is a common complication after blunt trauma to the chest. Imaging plays an important role in the diagnosis of sub-pleural hematoma. Conventional radiography is typically used for initial imaging examination, even if CT is to be performed. The role of ultrasound in diagnosis of sub-pleural hematoma has been emphasized (1).

A therapeutic thoracentesis in this case could have been used as the first line therapy. However, we used a conservative approach and the final result was excellent. Thoracentesis is used to remove a larger volume of pleural fluid or pleural hematoma to relieve symptoms, such as shortness of breath. In most cases, thoracentesis is performed without complications. When complications do occur, they are usually minor and resolve on their own or are easily treated. Potential complications include pain and discomfort, risk of bleeding from puncture of intercostal arteries, risk of infection, pneumothorax or lung collapse.

Pneumothorax is a symptomatic complication in approximately less than 5% of thoracentesis procedures (2), but could be less when performed with the assistance of ultrasound imaging (3). Recent data support the importance of operator expertise and the use of ultrasound in reducing the risk of iatrogenic pneumothorax (4). Pneumothoraces that do occur are usually small and resolve on their own; however, about one-third of pneumothoraces become large, continue to expand, or cause shortness of breath. In these patients a catheter or chest tube is placed through the skin into the pleural space to withdraw the air.

In conclusion, thoracenteses may have a very low complication rate. However, the current clinical guidelines for hematoma thoracentesis in pediatrics may not reflect evidence-based best practices and conservative management is a more appropriate approach.

## References

[1] JonesPWMoyersJPRogersJTRodriguezRMLeeYCLightRW. Ultrasound-guided thoracentesis: is it a safer method? Chest 2003; 123 (2): 418– 23. 1257636010.1378/chest.123.2.418

[2] AultMJRosenBTScherJFeinglassJBarsukJH. Thoracentesis outcomes: a 12-year experience. Thorax 2015; 70 (2): 127– 32. 2537854310.1136/thoraxjnl-2014-206114

[3] BarnesTWMorgenthalerTIOlsonEJHesleyGKDeckerPARyuJH. Sonographically guided thoracentesis and rate of pneumothorax. J Clin Ultrasound 2005; 33 (9): 442– 6. 1628126310.1002/jcu.20163

[4] CanteyEPWalterJMCorbridgeTBarsukJH. Complications of thoracentesis: incidence, risk factors, and strategies for prevention. Curr Opin Pulm Med 2016; 22 (4): 378– 85. 2709347610.1097/MCP.0000000000000285PMC8040091
